# Disc deformation as a potential biomarker of nonspecific low back pain

**DOI:** 10.1038/s41598-025-14366-5

**Published:** 2025-08-05

**Authors:** Kerstin M. Lagerstrand, H. Hebelka, H. Brisby, C. Waldenberg

**Affiliations:** 1 Institute of Clinical Sciences, Sahlgrenska Academy, University of Gothenburg, Gothenburg, Sweden; 2 Department of Biomedical Engineering and Medical Physics, Sahlgrenska University Hospital, Gothenburg, Sweden; 3 Department of Radiology, Sahlgrenska University Hospital, Region Västra Götaland, Gothenburg, Sweden; 4 Department of Orthopedics, Sahlgrenska University Hospital, Gothenburg, Sweden; 5 MR-center, Sahlgrenska University Hospital, Bruna straket 13, Göteborg, SE-400 36 Sweden

**Keywords:** Nonspecific LBP, Low back pain, Intervertebral disc, MRI, Deformation, Biomechanics, Discography, Medical research, Biomarkers, Diagnostic markers

## Abstract

Nonspecific low back pain (nLBP) lacks clear biomarkers, making diagnosis challenging. The disorder is commonly associated with annular fissures, which are hypothesized to trigger nerve endings to signal pain during disc deformation. Although symptoms worsen with load, MRI is typically performed in a relaxed state. Herein, a novel loading-based MRI method for non-invasive quantification of disc deformation was applied on 76 discs from 28 nLBP-patients to determine whether specific deformation patterns reflect fissures and pain using computer tomography and low-pressure discography during the same day as references. Discs without fissures and those with posterior fissures only displayed compression of annulus fibrosus posteriorly and expansion anteriorly, while discs with fissures both anteriorly and posteriorly and those with severe fissuring displayed elevated deformation and larger within-group variance (*p* = 0.001–0.04). Notably, discs with elevated anterior and low posterior deformation were more likely pain-signaling, whereas those with elevated posterior and low anterior deformation were often non-pain-signaling. At model probability thresholds below 0.38, all discs were correctly identified as non-painful and above 0.65, 86% were correctly identified as pain-signaling. Conclusively, this study provides in vivo evidence of a close interplay between disc deformation, fissures and pain, supporting the hypothesis that disc deformation contributes to nLBP.

**Supplementary Information:**

The online version contains supplementary material available at 10.1038/s41598-025-14366-5.

## Introduction

Diagnosing patients with nonspecific low back pain (nLBP) is challenging due to the lack of precise biomarkers^1^. Magnetic resonance imaging (MRI) provides superior contrast resolution for the intervertebral discs, spinal cord, nerve roots as well as the ligaments and soft tissues around the spine, making it a powerful tool in the evaluation of nLBP. However, the images often fails to reveal a clear cause, leading to potential misinterpretation of findings^2^. This may result in ambiguous treatment plans, unnecessary and inappropriate surgeries, increased patient suffering, and high societal costs^1^. Hence, both patients and society would benefit from improved MRI methods that can accurately identify specific features within the LBP group^3^.

While the exact cause of nLBP, as the name implies, is often nonspecific, it is known to be associated with annular fissures in the intervertebral discs^4,5^. Disc degeneration and increased stress on the discs are closely linked to annular fissuring, which can lead to structural weaknesses of the disc^6^. The weakening of the disc may be associated with altered biomechanical properties and micro-instabilities in the motion segment^7^. Consequently, the deformation of these affected discs may be altered and cause pain-signaling from nerve endings at the outer part of the annulus fibrosus.

Most individuals with nLBP experience aggravated symptoms during mechanical stress, yet MRI is conducted in a relaxed supine position^8–10^. Portable devices can be utilized in the MRI scanner to load the spine during the examination. This mechanical stress has been shown to impose changes in the discs, e.g., bulging and change of height^8,10,11^and to aggregate concordant pain in individuals with nLBP^9–16^. We have previously shown that deformation of the discs can be measured in detail by analyzing the local volume changes captured by comparing MRI scans obtained with and without spinal loading^17^. This technological advancement may enhance our understanding of how annular fissuring weakens the disc and the disrupted structural integrity is associated with changes in biomechanical properties. It is also plausible that the observed deformation pattern could be a biomarker of LBP.

Accordingly, this study aimed to determine whether disc deformation is linked to annular fissuring and has the potential to phenotype/target the pain in patients with nLBP. The aim was investigated by loading-based MRI to assess disc deformation, with computed tomography (CT) and low-pressure discography as references of annular fissuring and pain provocation.

## Methods

### Study sample

All experiments were performed in accordance with the Declaration of Helsinki and approved by the Institutional Review Board (Protocol code Dnr 366-07/2007 and Dnr 2022-03018-02/2022). Informed consent was obtained from all participants.

The study sample comprised 28 patients with nLBP (mean age 45 years, range 28–63, 57% women), who had previously been enrolled in a prospective MRI-discography study that explored high-intensity zones (HIZ) in association with spinal loading and pain provocation at discography^11^. The original MRI-discography study included 62 nLBP patients who had been referred for preoperative lumbar discography and had experienced nLBP for at least 6 months, which was severe enough to warrant consideration for surgery. Further, patients with allergies to contrast media and contraindications to MRI were not included.

To address the specific aim of this study, only patients who had a full dataset consisting of MRI examination in the supine position with and without spinal loading, low-pressure discography, and CT, were included.

### Examination protocol

Each patient had been examined during the same day with MRI, followed by low-pressure discography and CT.

#### MRI examination

The MRI examinations were performed on a 1.5T scanner (Siemens Magnetom Symphony Maestro Class, Erlangen, Germany) and included standardized sagittal T1- and T2-weighted imaging covering L1-S1 as well as axial T2-weighted imaging at targeted levels (Table 1).

**Table 1:** Acquisition and reconstruction parameters for MRI and CT scans.

Parameter	T1-weighted MRI (TSE)^a^	T1-weighted MRI (SE)^a^	T2-weighted MRI (TSE)^a^	T2-weighted MRI (TSE)^a^	CT^b^
Plane of imaging	Sagittal	Axial	Sagittal	Axial	Sagittal, Axial
TR (ms)	448–692	400–645	4000–5160	3800–7870	
TE (ms)	11–12	15	120–124	116–119	
Echo train	9	1	21	25	
Slice thickness (mm)	4	4	4	4	0.75 (reconstructed)
Gap between slices (mm)	0.4	0.4	0.4	0.4	
Averages (NEX)	2–4	2	2	3–4	
Bandwidth (Hz/pixel)	200	100	190	190	
Flip angle (degrees)	147–149	90	150	150	
Acquisition matrix	512 × 256	256 × 135	512 × 256 (448 × 224)^*^	256 × 126	
Reconstruction matrix	512 × 512	384 × 512	512 × 512 (448 × 448)*	360 × 512	512 × 512
FOV (mm²)	300 × 300	135 × 180	300 × 300	127–138 × 180–196	162 × 162
Convolution kernel					B45s

^a^MRI: 1.5T Siemens Magnetom Symphony Maestro Class (Erlangen, Germany). ^b^CT: Siemens Somatom Sensation 16-slice (Erlangen, Germany). *Matrix sizes for one patient with differing scan parameters.

T1W = T1-weighted; T2W = T2-weighted; MRI = magnetic resonance imaging; CT = computed tomography; SE = spin echo; TSE = turbo spin echo

**Fig. 1:**
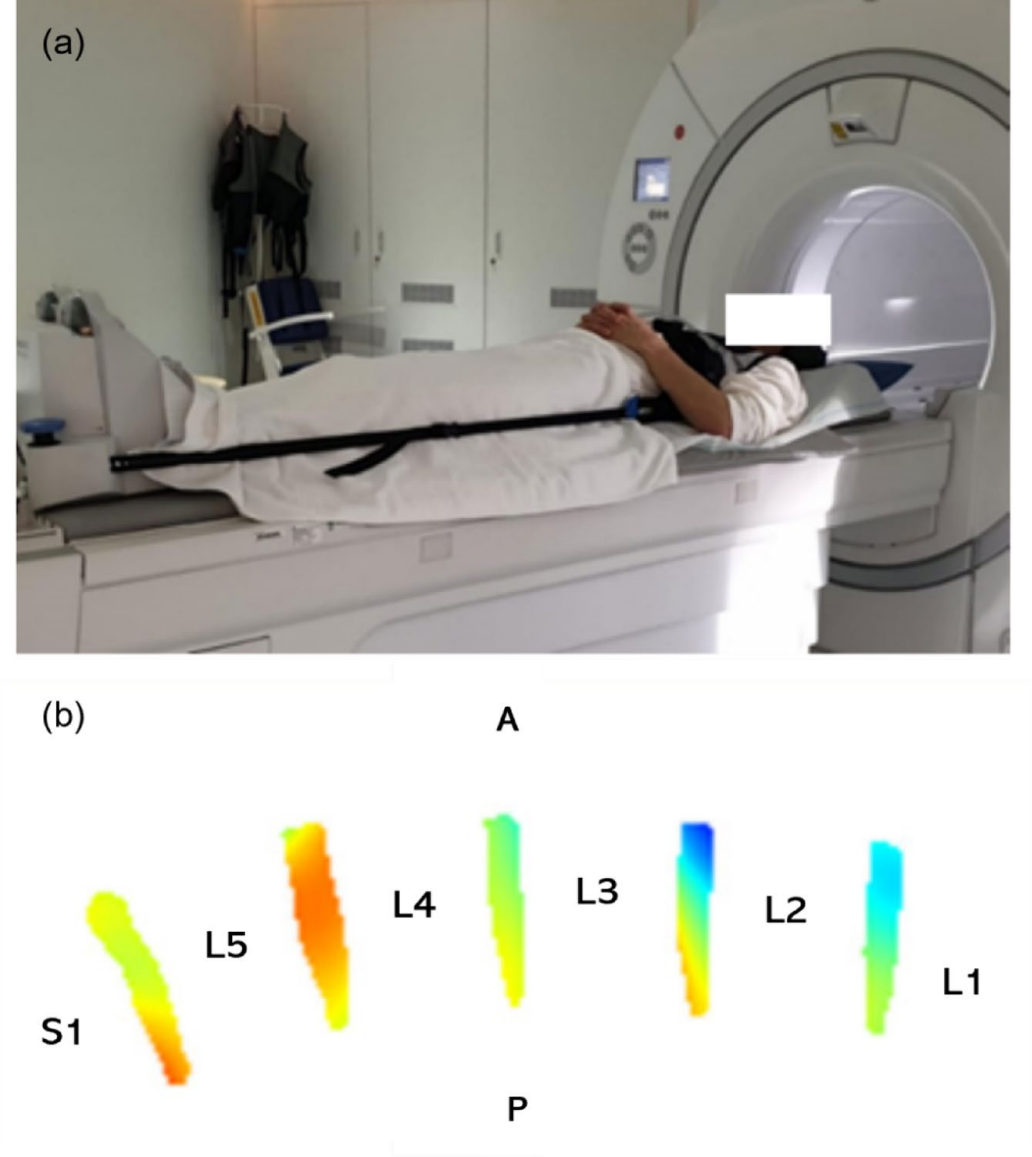
(**a**) A participant lies in the supine position on the tabletop of a whole-body MRI scanner with the Dynawell Diagnostics^®^ compression device applied to axially load the spine during the examination. (**b**) The deformation of the intervertebral discs during the spinal loading can be quantified by registering MR images with and without spinal loading to calculate the local compression (red) and expansion (blue) in terms of the Jacobian determinant of the registration deformation field. Written informed consent has been obtained from the participant for permission to use the actual image.

The patients were also imaged during spinal loading, after approximately 15 min load, using the same sagittal T2-weighted scan as reported above. A portable compression device (Dynawell Diagnostics^®^) was used to apply load on the spine during MRI (Fig. 1)^9–13,18,19^. The device was adjusted to 50% of the body weight to simulate typical macroscopical disc alterations generated in an upright standing position^9,10,18,20,21^.

#### Pain provoked by discography

Pressure-controlled discography was conducted after the MRI examination, without sedation, in all participants. The disc selection was based on combined findings from clinical assessment and prior MRI in the lumbar levels.

In this study, all included discs were re-classified using the operational criteria for pathogenic pain during discography, as stated by the International Spine Intervention Society (IPSIS)^22^. Accordingly, pain considered ”true” discogenic was classified as the pain produced by stimulating the target disc, which was reproducible, concordant with daily pain, rated ≥ 7/10 on the numerical rating scale (NRS), at a pressure ≤ 50 psi above the opening pressure, and further it was not painful to stimulate at least one adjacent disc. Discs with no pain was defined as those with pain-negative discograms, i.e., discs that either did not induce pain or elicited a pain response unfamiliar to the patient during discography. In addition, the number of discs that did not meet the operational criteria for pathogenic pain during discography was recorded.

#### CT examination

CT examination of the lumbar spine was performed ~ 30–60 min after the discography procedure using a 16-slice CT scanner (Siemens Somatom Sensation, Erlangen, Germany) with 180 mg/ml contrast media (Omnipaque, GE Healthcare, Oslo, Norway); see Table 1 for more CT scan parameters. After the examination, multiplanar reconstructions with 1 mm thickness were generated in sagittal, coronal, and axial planes, providing a detailed visualization of contrast media distribution across all planes.

### Quantification of the disc deformation

The quantification of the disc deformation has been described in detail by Johansson et al.^17^ and in principle herein. For each patient, MR images with and without loading were registered to a common spatial volume using Elastix (open source software built upon ITK^23^. Disc deformation was then calculated in five midsagittal slices as the Jacobian determinant of the registration deformation field (Fig. 1). This metric quantifies local volume changes, where positive values indicate expansion, while negative values indicate compression. Typical deformation patterns in subregions along the anterior-posterior direction were then determined, highlighting regions of compression and expansion within the discs. To allow for comparison of deformation patterns across discs, deformation values were normalized by setting the deformation at the disc center to zero.

“Normal deformation patterns” were determined for discs without annular fissures. These patterns were compared with those for discs with annular fissures of varying extent, including different grades and locations. Since outer anterior and posterior regions of the annulus fibrosus may contain nerves capable of signaling pain, the deformations in these subregions were compared between discs with no pain and those who signaled pain during provocation. For comparison, the association between discogenic pain and annular fissuring was also determined.

### Radiological classifications of annular fissures and disc degeneration

A senior radiologist specializing in musculoskeletal care had previously classified all included discs using a modified Dallas Discogram Description (DDD)^24^ scale as follows: score 0 – no fissures and intact nucleus, score 1 – existence of fissures at inner 2/3rd of annulus fibrosus, score 2 – fissures extending beyond outer 2/3rd of annulus, and score 3 – fissures extending beyond outer annulus fibrosis. Discs with confirmed fissures were further classified based on whether the fissure was delimitable or not^25^. A delimitable fissure was defined as a clearly defined fissure or several such fissures. In contrast, a non-delimitable fissure had severely disrupted annulus fibrosus, with < 50% of the outer annulus continuously intact. In addition, the position of the fissures was determined. Fissures were classified as anterior if they involved subregion 1, and as posterior if they involved subregion 5, regardless of whether they extended beyond those areas. Lateral fissures were defined as those confined to subregions 2–4. Based on these fissure classifications, the included discs were sorted into the following categories: (1) no fissures, (2) posterior fissures, (3) anterior and posterior fissures, and (4) severe fissuring (< 50% continuously intact outer third annulus fibrosus)^25,26^.

Further, disc degeneration had been classified using the Pfirrmann Classifcation^27 ^and the presence of HIZ^28^ had been identified in the MR images. These MRI findings were included in the present study for reference.

### Statistical analysis

All statistical calculations were performed with MATLAB R2022b. The available data determined the present sample size. Wilcoxon rank-sum and Levene’s test were used with *p* < 0.05 to determine group differences and equality of variances. A binary logistic regression model was used to explore possible associations between deformation and Fissure category or pain, where the strength of the model was described using receiver operating characteristics (ROC), including the area under the curve (AUC). Separate models were fitted for different groups, using discs without fissures as reference. Discs with only anterior or lateral fissures were excluded from the fissure-deformation analysis due to low sample size but were included in the pain-deformation analysis. Odds ratios (ORs), 95% confidence intervals (CIs), and p-values were reported for each model. A probability surface plot was generated with contour lines to visualize deformation associated with more confident classifications.

## Results

### Study sample

**Fig. 2:**
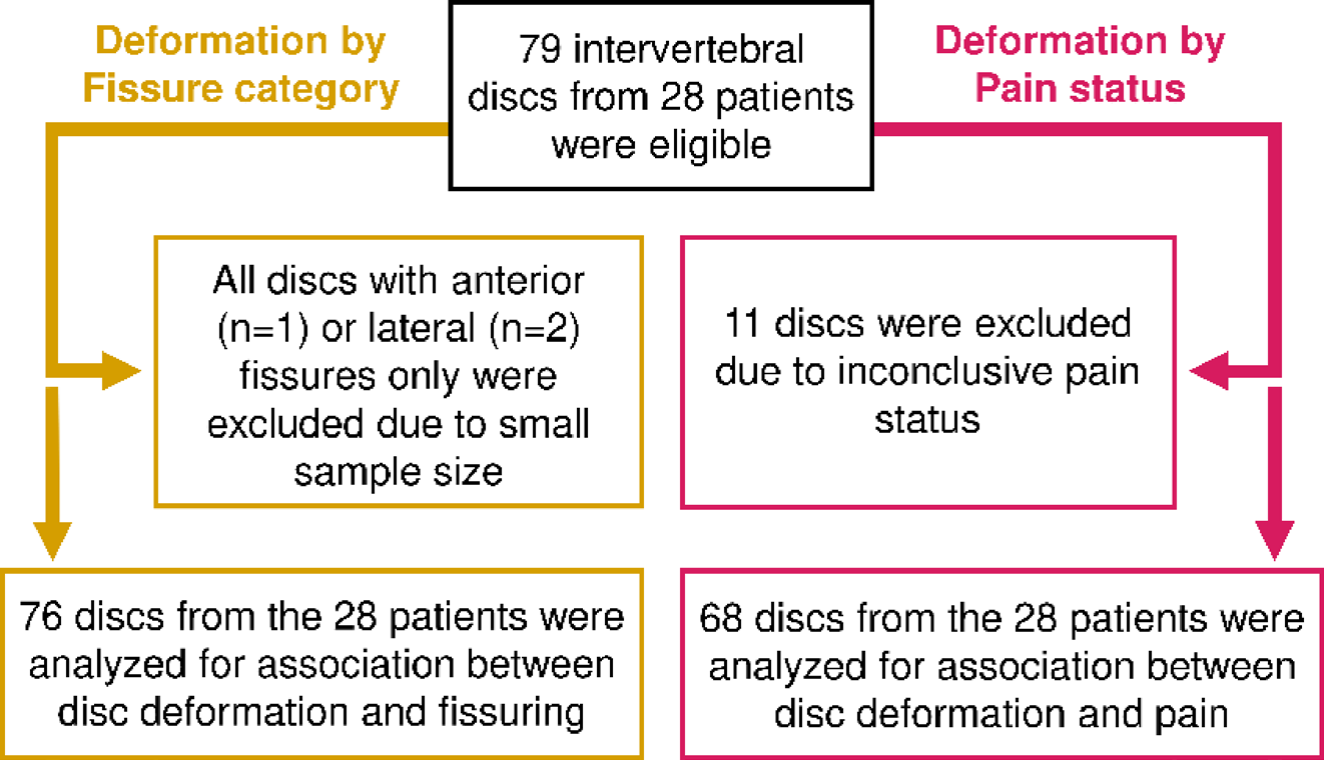
Flow chart displaying inclusion and exclusion.

A total of 28 nLBP patients [mean age: 45.4 ± 9.4 years; 16 (57%) women] with 79 injected discs were included (Fig. 2). The number of injected discs per patient ranged from 1 to 4. Of these discs, 68 met the operational criteria for pathogenic pain during discography)^22^ and were accordingly separated into discogenic (44%) and non-discogenic pain (42%). In the remaining discs (14%), the operational criteria for pathogenic pain could not be determined since discography had not been performed in at least one of the adjacent discs. These discs were included in the deformation-fissure analysis but excluded from the deformation-pain analysis. Furthermore, no significant differences in age or gender distribution were observed between patient subgroups classified as discogenic and no discogenic pain [45.4 ± 9.5 years, 40% women vs. 46.0 ± 9.9 years, 36% women].

Table 2 presents the radiological classification parameters per disc for both the CT discograms and MRI. A more detailed table is provided in the supplementary data (Table S1). Among the included discs, 14 displayed no fissures, while the rest exhibited fissures that were posterior (*n* = 43), anterior (*n* = 1), lateral (*n* = 2), both anterior and posterior (*n* = 7), or had severe fissuring (*n* = 12).

**Table 2:** Disc classifications on both CT discograms and MRI for all discs, as well as for discs categorized according to the operational criteria for pathogenic pain during discography.

Variable	Total	Discogenic pain	No discogenic pain	Inconclusive pain status^†^
	*n* = 79	*n* = 35	*n* = 33	*n* = 11
**Spinal level**				
L1-L2	1	0	1 (100%)	0
L2-L3	10	2 (20%)	8 (80%)	0
L3-L4	27	8 (30%)	14 (52%)	5 (19%)
L4-L5	26	14 (54%)	6 (23%)	6 (23%)
L5-S1	15	11 (73%)	4 (27%)	0
**Fissure category**				
No fissure	14	1 (7%)	13 (93%)	0
Anterior*	1	1 (100%)	0	0
Posterior	43	19 (44%)	14 (33%)	10 (23%)
Lateral*	2	1 (50%)	1 (50%)	0
Anterior + Posterior	7	2 (29%)	5 (71%)	0
Severe fissuring	12	11 (92%)	0	1 (8%)
**Pfirrmann classification**				
Grade 1	0	0	0	0
Grade 2	8	0	8 (100%)	0
Grade 3	33	14 (42%)	15 (45%)	4 (12%)
Grade 4	36	19 (53%)	10 (28%)	7 (19%)
Grade 5	2	2 (100%)	0	0
**High-intensity zone**				
None	34	10 (29%)	22 (65%)	2 (6%)
Anterior	2	2 (100%)	0	0
Posterior	42	23 (55)	11 (26)	8 (19)
Anterior + Posterior	1	0	0	1 (100)

Note. Except where indicated, values refer to the number of intervertebral discs, with percentages in parentheses. *Fissure categories marked with an asterisk were excluded from the analysis of the association between annular Fissure category and disc deformation due to low sample size. SD = standard deviation. ^†^Inconclusive according to the operational criteria for pathogenic pain during discography^22^.

### Association between disc deformation and fissures

**Fig. 3:**
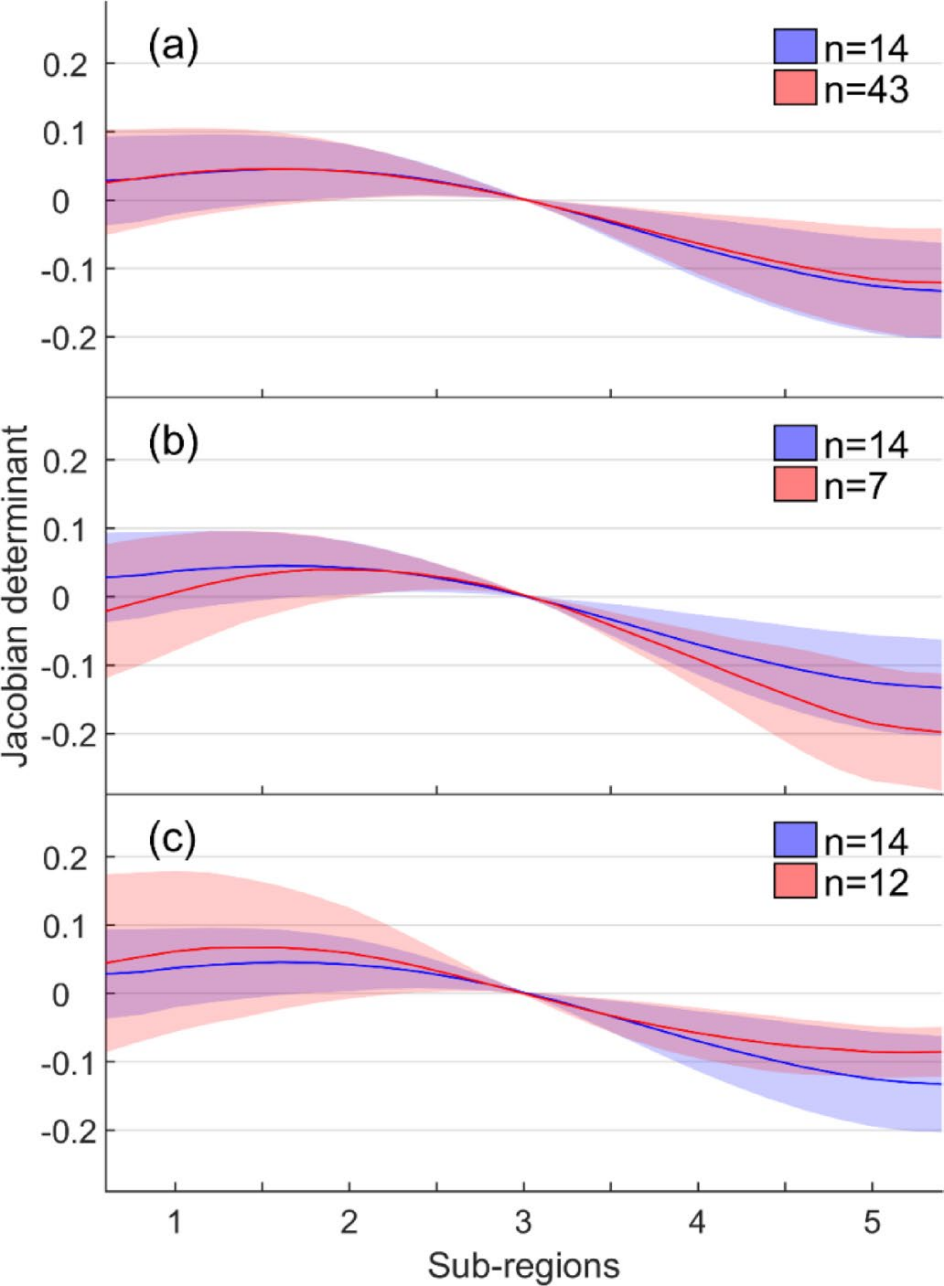
Disc deformation, quantified in terms of the Jacobian determinant, without fissures (blue) and with (**a**) posterior fissures only, (**b**) anterior and posterior fissures, and (**c**) severe fissuring (red). The Jacobian determinant reflects local volume change, where values above zero indicate relative expansion, and values below zero indicate relative compression. To enable comparison across discs, the values were centered so that the deformation at the disc center (subregion 3) is zero. The x-axis represents five subregions along the anterior–posterior axis of the disc, from anterior (1) to posterior (5). Shaded areas indicate ± 1 standard deviation from the group mean deformation. Discs with only anterior (n = 1) or lateral fissures (n = 2) were excluded due to low sample size.

Figure 3 displays the disc deformation for the different fissure categories. Please see Table S1 in the supplementary data for deformation values per individual discs. In general, discs without fissures displayed compression of the annulus fibrosus posteriorly (Jacobian determinant < 0) and expansion anteriorly (Jacobian determinant > 0) (Fig. 3). Discs with posterior fissures only displayed similar deformation pattern as those without fissures (Fig. 3a). On the contrary, discs with fissures both anteriorly and posteriorly (Fig. 3b) and those with severe fissuring (Fig. 3c) displayed greater deformation. In 21% of these cases, the deformation was more than two standard deviations larger than the mean deformation in discs without fissures.

**Fig. 4:**
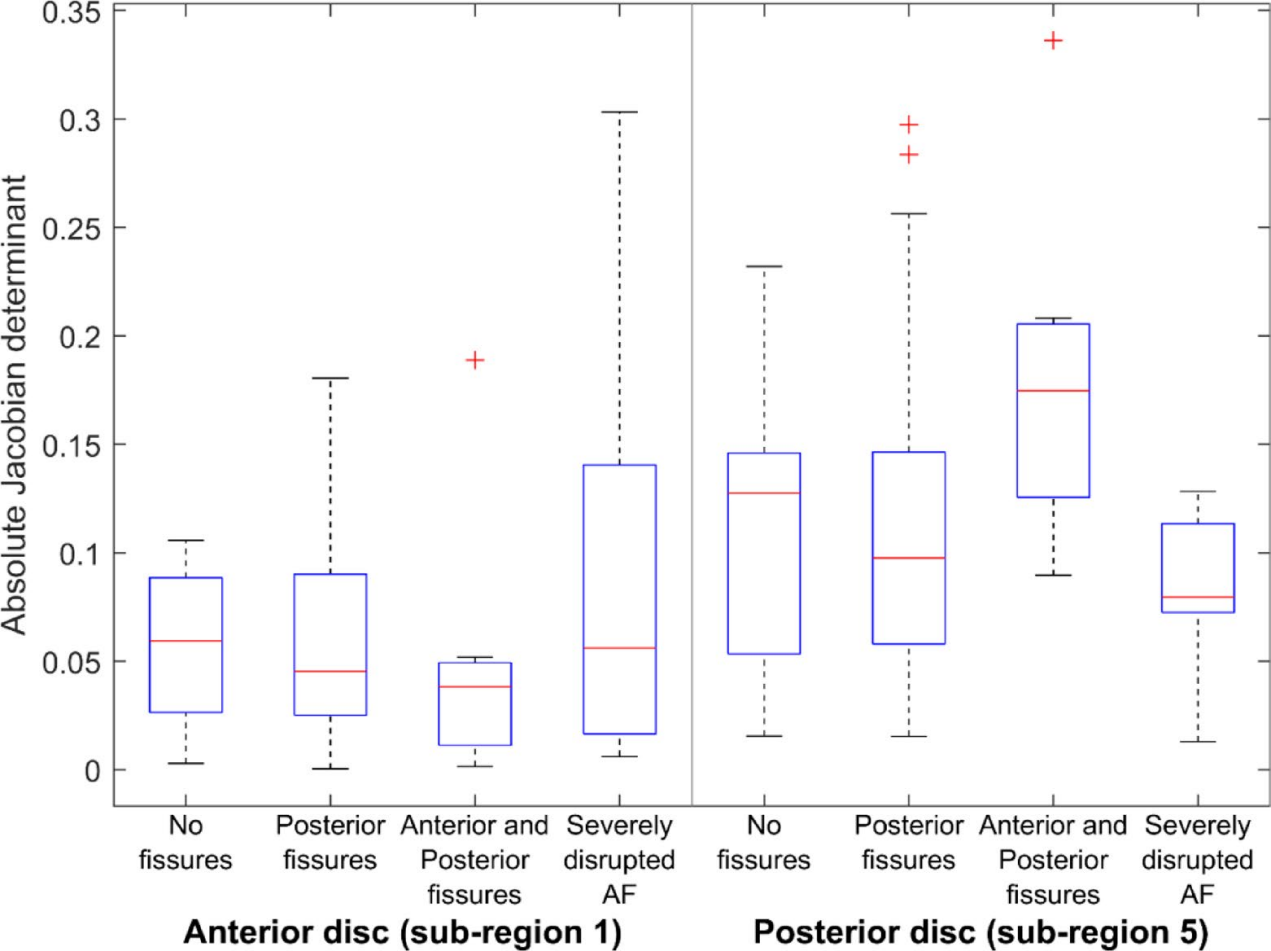
Box plots displaying the absolute Jacobian determinant in disc sub-region 1 (anterior) and sub-region 5 (posterior), grouped by annulus fibrosus (AF) Fissure category. Deformation values were centered so that the deformation at the disc center (subregion 3) is zero, and absolute values are used to reflect the magnitude of deviation regardless of disc compression or expansion. The absolute Jacobian determinant is presented as |J − 1|, where |J − 1| = 0 indicates no deformation.

Side-by-side comparisons of deformation patterns in different subregions of the disc along the anterior-posterior direction revealed no significant differences between groups of discs without fissures and those with posterior fissures only or combined anterior and posterior fissures (Fig. 4; Table 3). However, discs with severe fissuring showed significantly higher deformation variance in subregions 1 and 5 compared to discs without fissures.

**Table 3:** Comparison between groups of intervertebral discs with different categories of annular fissures, using the p-value of the Wilcoxon signed-rank test for differences among group medians and levene’s test for equality of variances among groups.

	Sub-region 1		Sub-region 2		Sub-region 3		Sub-region 4		Sub-region 5	
	W	L	W	L	W	L	W	L	W	L
**Group 1**	0.993	0.218	0.732	0.983	n/a	n/a	0.475	0.956	0.547	0.767
**Group 2**	0.314	0.441	0.526	0.920	n/a	n/a	0.351	0.949	0.168	0.872
**Group 3**	0.521	**0.004**	0.700	0.087	n/a	n/a	0.456	0.667	0.095	**0.035**

Group 1: discs without fissures vs. discs with posterior fissures.

Group 2: discs without fissures vs. discs with anterior and posterior fissures.

Group 3: discs without fissures vs. discs with severe fissuring (<50% of the outer third annulus fibrosus continuously intact).

n/a: Test not applicable since this region was used for normalization. W: Wilcoxon signed-rank test.

L: Levene’s test. Significant differences are determined at p<0.05 and indicated with bold font.

**Fig. 5:**
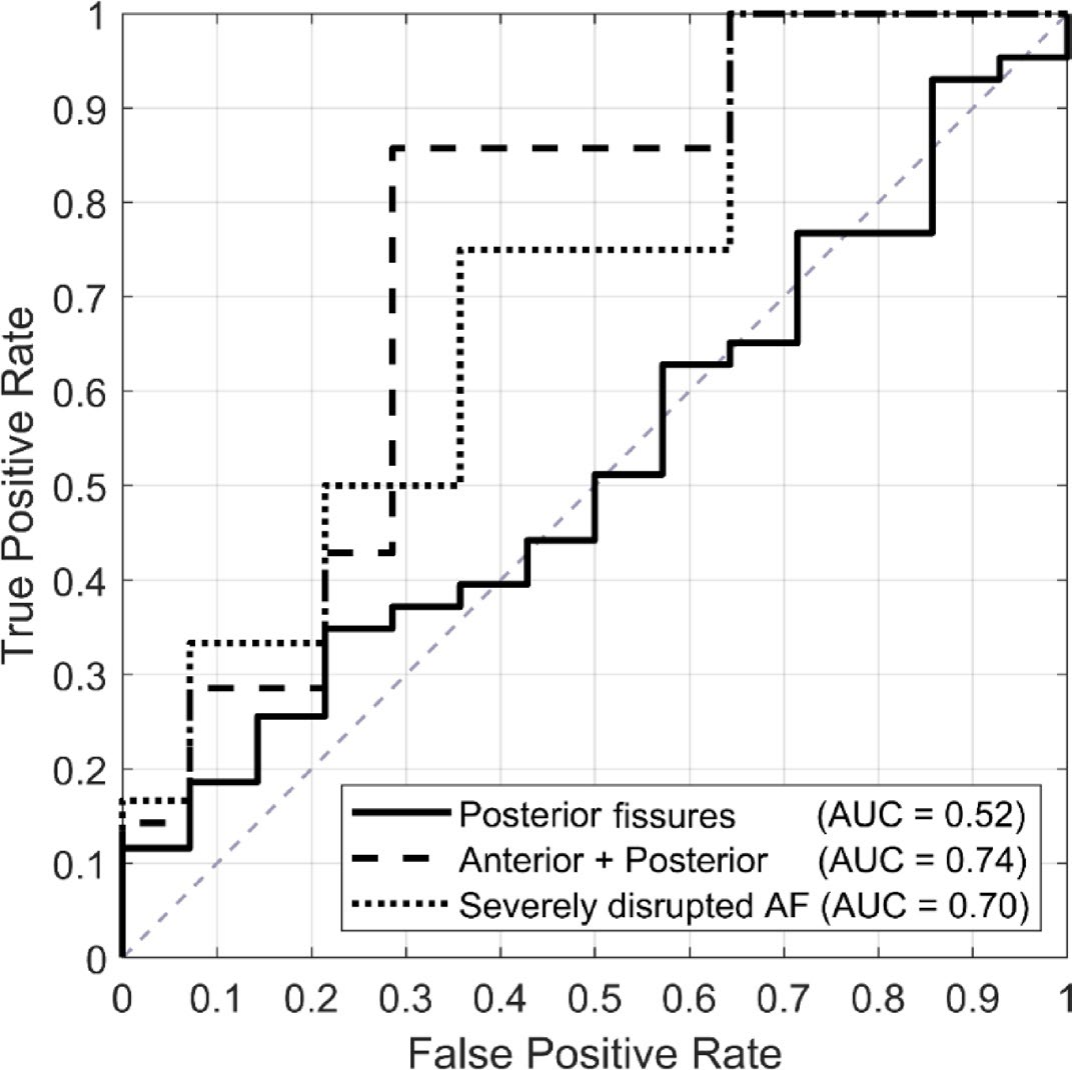
The curves show the separability between fissured and non-fissured discs based on anterior and posterior deformation. Each curve represents a separate logistic regression model, comparing different fissure categories (posterior only, anterior + posterior, or severe) against discs without fissures. Discs with more extensive fissuring (anterior + posterior fissures or severe fissuring) showed better separability from non-fissured discs than those with posterior fissures only.

The analysis of the association between disc deformation and annular fissuring showed that discs with posterior fissures only could not be separated from non-fissured discs based on the deformation pattern (Fig. 5). Neither anterior and posterior fissures or severe fissuring were significantly associated with the disc deformation (Table 4). Table 4 further shows that discs with posterior fissures only and those with severely disrupted annulus fibrosus more likely displayed increased anterior deformation and reduced posterior deformation compared to discs without fissures. In contrast, discs with both anterior and posterior fissures more likely showed the opposite pattern, with reduced anterior and markedly increased posterior deformation.

**Table 4:** Association between disc deformation and fissure category.

Metric	Posterior fissures	Anterior and posterior fissures	Severe fissuring
n (fissure)	43	7	12
n (no fissure)	14	14	14
AUC	0.52	0.74	0.70
Anterior OR (p-value) [95% CI]	7.49 (*p* = 0.79) [< 0.01, 1.74 × 10¹⁰]	0.01 (*p* = 0.65) [< 0.01, 2.17 × 10⁷]	5.65 × 10³ (*p* = 0.26) [< 0.01, 2.19 × 10¹⁰]
Posterior OR (p-value) [95% CI]	0.20 (*p* = 0.71) [< 0.01, 1.05 × 10³]	4.10 × 10⁵ (*p* = 0.11) [0.06, 2.74 × 10¹²]	< 0.01 (*p* = 0.11) [< 0.01, 30.34]

AUC = Area Under the Receiver Operating Characteristics (ROC) Curve; OR = Odds Ratio; CI = Confidence Interval. n denotes the number of discs for the specific group. Confidence intervals are reported at the 95% level.

### Disc deformation as a potential biomarker of pain

**Fig. 6:**
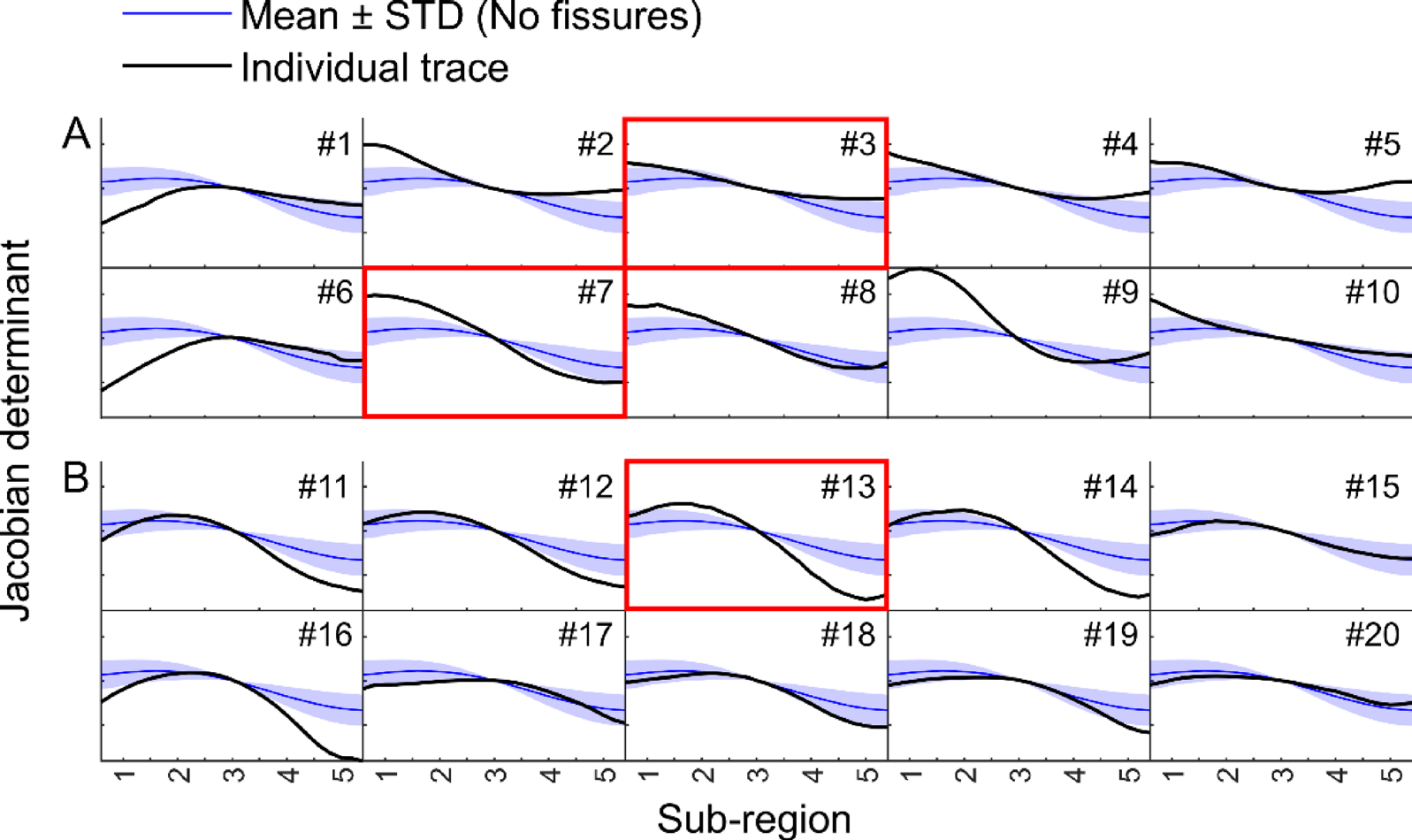
Disc deformation patterns for the 10 discs most confidently classified as painful (**A**) and the 10 discs most confidently classified as non-painful (**B**). Red boxes indicate misclassified cases: two false positives in A (#3 and #7), and one false negative (no-pain) in B (#13). Each panel shows the deformation profile of one disc (black curve) relative to the mean ± 1 standard deviation (STD) of discs without annular fissures (blue shaded area). Values are centered so that deformation at the disc center (sub-region 3) is zero. The x-axis spans five sub-regions along the anterior–posterior axis of the disc, from anterior (1) to posterior (5).

**Fig. 7:**
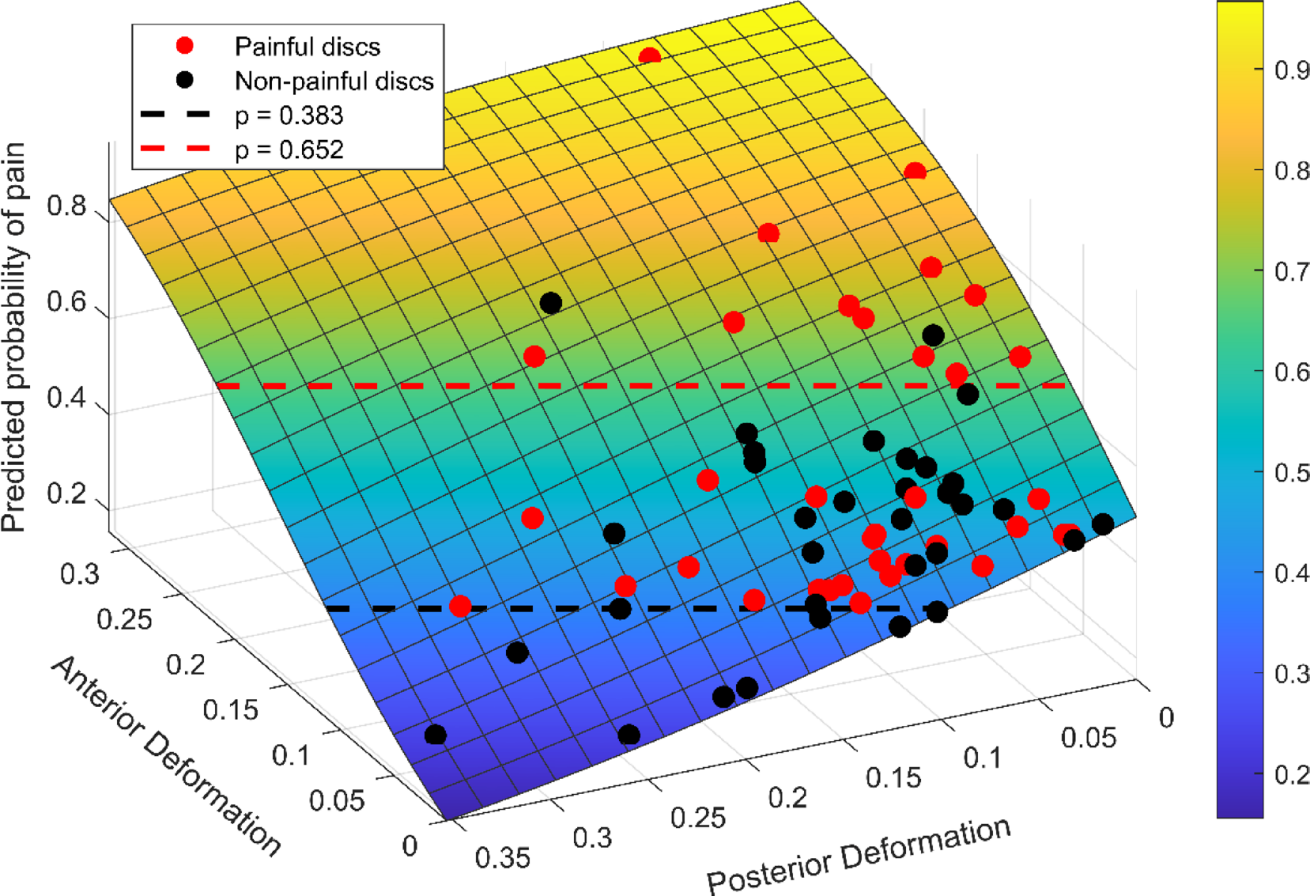
Surface plot displaying the model-predicted likelihood of discogenic pain based on anterior and posterior disc deformation, quantified in terms of the absolute Jacobian determinant. Red and black dots represent painful and non-painful discs, respectively. The plot illustrates that discs with greater anterior deformation and minimal posterior deformation tend to be painful, whereas those with greater posterior deformation and minimal anterior deformation tend to be non-painful. The contour lines also show that the same likelihood of pain can arise from different anterior and posterior deformations, reflecting the combined influence of both regions. Dashed contour lines mark two thresholds where the model performed with high certainty (*p* = 0.383 and 0.652).

**Fig. 8:**
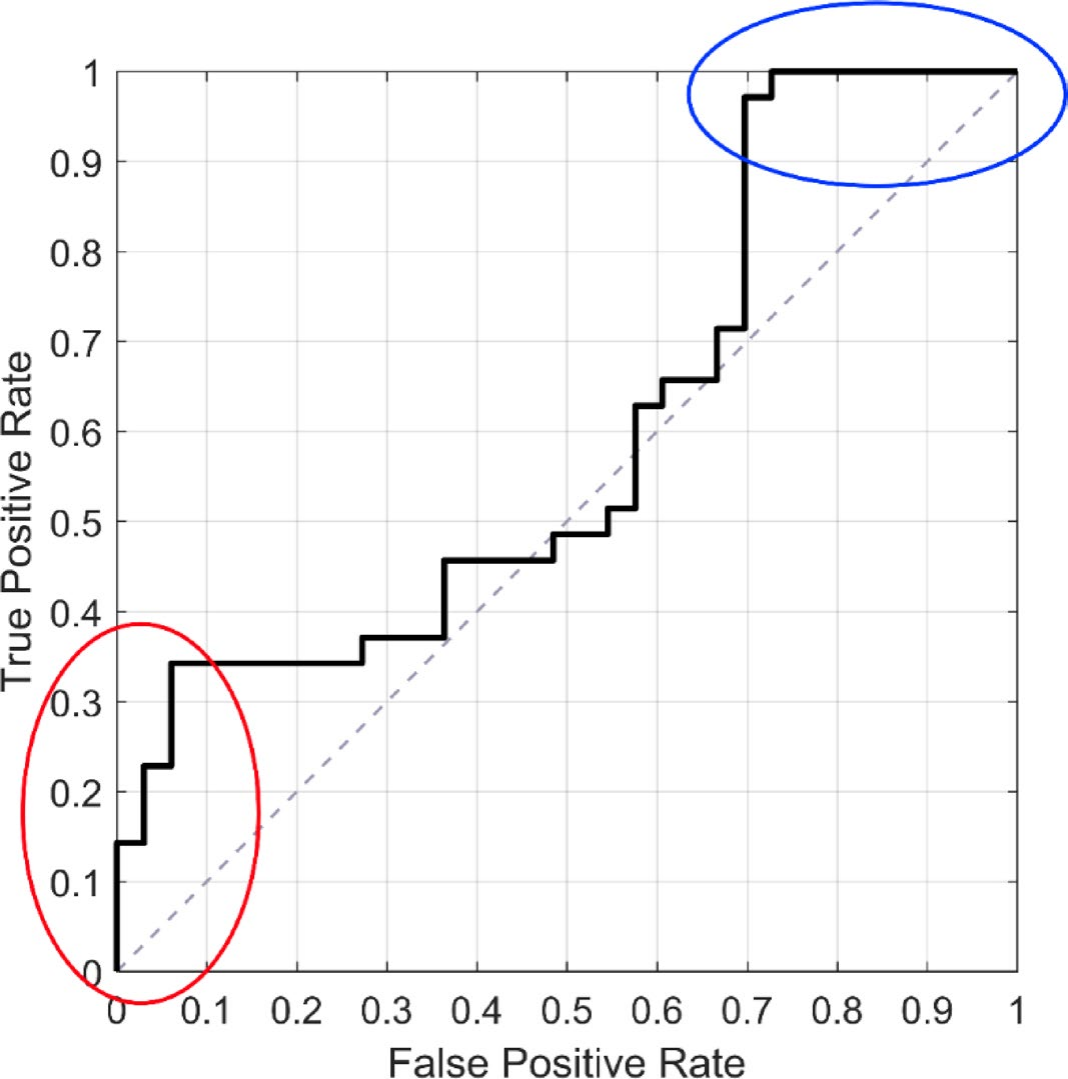
The ROC-curve for the prediction of discogenic pain using disc deformation as a potential biomarker shows that a specific combination of deformation patterns can identify “true” pain (red circle) and no pain (blue circle) with high certainty.

Discs with high anterior and low posterior had a higher likelihood of being classified as pain signaling, whereas those with low anterior and high posterior deformation were more often classified as non-pain signaling. (Figs. 6 and 7). Notably, a subset of non-painful discs showed this low anterior - high posterior deformation pattern and were all correctly identified by the model. However, discs with intermediate deformation showed mixed classification outcomes, making the pain status difficult to distinguish based on deformation alone. This trend was reflected in the ROC analysis (Fig. 8), which illustrated that specific deformation patterns corresponded to high-confidence predictions, while intermediate patterns did not. Moreover, probability thresholds were identified that further support the relationship between disc deformation and pain status: discs with predicted pain probabilities < 0.38 were all correctly classified as non-painful (*n* = 9), while those with probabilities > 0.65 were correctly classified as painful in 12 out of 14 cases. In a complementary analysis, the 10 discs most confidently classified as painful and the 10 most confidently classified as non-painful, representing the extremes of the model’s probability range, were examined (Fig. 6). Of these 20 discs, 17 were correctly identified, with two false positives and one false negative.

Fissure category could not explain discogenic pain as effectively as the deformation pattern (Table 2). While pain provocation was more often registered in severely fissured discs, pain signaling was also registered for a relatively large number of discs with posterior fissures only. Among discs without fissures, pain provocation was registered in 1 of 14 discs (7%). In contrast, pain during provocation was observed in 19 of 43 discs (44%) with posterior fissures only, 2 of 7 discs (29%) with both anterior and posterior fissures, and 11 of 12 discs (92%) with severe fissuring. Please see Table S1 in the supplementary data for details.

## Discussion

Present study demonstrates the value of loading-based MRI for nLBP and evaluates how the intervertebral discs deform in response to the increased mechanical stresses depending on annular fissuring and discogenic pain. While discs with posterior fissures only showed normal deformation and weak association with the pain, extensive fissuring with deviant deformation patterns was more frequently observed in “painful discs”. These findings suggest that deviant deformation patterns may serve as an indicator of underlying micro-instability in the motion segment^7^, where nerve endings at the outer part of the annulus fibrosus are triggered to signal pain during the increased mechanical stress. It is too early draw conclusions on clinical application at this stage, but such precise biomarkers may streamline the diagnosis of non- nLBP, thereby better guide the decision-making for managing this large patient group; ultimately improving patient care and reducing the healthcare burden.

Present study also moves the current research front forward by presenting how the disc deformation is associated with annular fissuring in vivo. Discs with posterior fissures only could not be distinguished from intact discs based on the observed deformation, suggesting that such fissures exhibit similar deformation patterns as in structurally intact tissue. In contrast, discs with more extensive annular fissuring, including those with both anterior and posterior fissures and severe fissuring, displayed a different biomechanical response to mechanical stress, particularly in the anterior region. Even though the nature of the disc deformation seemed to be associated with fissure category, statistically significant differences between fissure categories could not be established, and the distributions showed wide, overlapping confidence intervals. This suggests that the observed disc deformation may depend on other factors, such as disc level, lumbar lordosis, and variations in fissure morphology, that warrant further investigation. Nevertheless, the findings indicate that disc deformation, particularly in the anterior region, may reflect loss of structural integrity in discs with more extensive fissuring. Increased anterior deformation appeared more common in these discs, suggesting that it may serve as an indicator of underlying structural damage.

While there might be value in identifying annular fissures as early manifestations of the degenerative cascade^29^, present findings suggest that the presence of fissures only cannot phenotype/target the origin of pain in patients with nLBP. Additionally, HIZs, which represent annular fissures consisting of vascularized granulation tissue, demonstrated limited utility in identifying discogenic pain. Although the presence of HIZ has been proposed as a diagnostic marker for nLBP^30^its clinical relevance has been debated over the years^30,31^. The findings herein further suggest that the concept of HIZ as a uniform indicator of pain may warrant reevaluation.

A key strength of this study was the access to a unique dataset, including MRI of nLBP patients in both “normal relaxed” and “loaded” states, as well as registrations of fissure category and discogenic pain. This set of data enabled the identification of typical deformation patterns that may have the potential to target pain-signaling discs in selected individuals. Based on the available dataset, we could also determine deformation thresholds in related to pain provocation. At the lower threshold, characterized by low anterior and higher posterior deformation, all discs were correctly identified as non-painful with no false positives. At the higher threshold, characterized by high anterior and low posterior deformation, 86% of the discs were accurately classified as “painful”. Although the findings strongly suggest that elevated anterior deformation may contribute to discogenic pain in selected discs, the observed disc deformation could not explain the pain in cases with less pronounced deformation patterns. This underscores the complexity of nLBP and the importance of interpreting disc deformation within a broader clinical context. Also, it should be noted that among the subset of discs with the most confident model predictions, two false positives and one false negative were observed, highlighting that even when deformation closely resembles painful or non-painful patterns, unexplored individual variability may have an underlying impact on the perceived pain experience.

The pathophysiology of discogenic pain depends on the sensory nerves^32^. Typically, these innervate the outermost third of the annulus^33^. However, nerve ingrowth into the inner layers of the disc can occur through annular fissures^34^. It has been hypothesized that the disrupted annular fibers allow inflammatory mediators to irritate nearby nerves, causing discogenic pain. This pain is especially aggravated in positions with elevated load on the spine^8–10^. At such positions, the mechanical stress may deform the annular fissures and trigger the nerves to signal discogenic pain, as shown herein.

As demonstrated herein, not all discs that signal pain during provocation demonstrate deformation patterns consistent with micro-instability and may therefore not benefit from spine-stabilizing procedures such as fusion surgery or core-based physiotherapy. The proposed loading-based MRI method, designed to detect such deformation patterns, could help clarifying subtypes of nLBP and identify a biomechanically driven discogenic pain phenotype. However, validation in large multicenter studies is needed to confirm the biomarkers reproducibility, robustness, and generalizability across populations and imaging systems before application in the clinical setting. Validation should include asymptomatic controls to distinguish true pathology from age-related degeneration. Including asymptomatic controls will further enhance the interpretation of the findings, strengthen the construct validity of the proposed biomarker, and refine its clinical utility in distinguishing discogenic pain from non-specific findings.

Provocative discography is a controversial diagnostic tool due to its invasiveness, subjectivity, and low specificity, as well as the potential for pressure transfer to adjacent discs, which can result in false positive pain responses. However, studies have reported that careful control of pressure and injection speed can mitigate these issues^35^. A systematic review by Manchikanti et al. supports the diagnostic accuracy of provocation discography if based on the IPSIS criteria, as used herein^36^. Similarly, Wolfer et al., through a meta-analysis of discography studies involving asymptomatic individuals, reported that provocation discography performed in accordance with these criteria was associated with a low false-positive rate^37^. However, provocation discography does not always reproduce the patient’s typical pain^38^which may partly explain the few misclassifications in our study. There are, however, concerns that invasiveness of the discography procedure may accelerate lumbar disc degeneration^39^. In contrast, the present loading-based MRI method provides a feasible and objective tool to non-invasively identify discs that may be pain generators in vivo^17^. It builds on an easily-handled compression device, a standardized scan protocol, and a well-established image registration software, features that support its potential clinical use. While the loading-based MRI method is designed to be relatively robust to typical image contrast variations, its performance across different scan settings remains to be confirmed.

It should also be noted that the small sample size, especially within subgroups of discs categorized by specific fissure types, reduced the statistical power of the analysis and increases the risk that non-significant findings are due to insufficient data rather than the absence of a true effect. Discs with anterior fissures were excluded from the deformation analysis due to low representation, which may have omitted relevant biomechanical patterns. Furthermore, fissures were classified in the mid-sagittal planes and, as such, did not capture structural variations in the lateral or superior-inferior directions. However, as fissures are believed to occur mainly along the anterior-posterior axis, the selected planes likely captured the most relevant biomechanical changes.

## Conclusions

This study provides in vivo findings of the interplay between annular fissuring and disc deformation and supports the hypothesis that the deformation of the disc is associated with discogenic pain. Discs with posterior fissures only displayed similar deformation patterns as discs without fissures, while discs with both anterior and posterior fissures and those with severe annular fissuring displayed deviant patterns with large within-group variance. Notably, discs with elevated anterior and low posterior deformation were more likely pain-signaling, whereas those with elevated posterior and low anterior deformation were often non-pain-signaling. The observed associations between the disc deformation and discogenic pain strongly suggest that specific deformation patterns may carry clinically relevant information. Future studies are encouraged to confirm the clinical value of this promising nLBP biomarker.

## Supplementary Information

Below is the link to the electronic supplementary material.

Supplementary Material 1

## Data Availability

The datasets generated during and/or analysed during the current study are available from the corresponding author on reasonable request.
